# Occurrence of Hepatitis E Virus in Pigs and Pork Cuts and Organs at the Time of Slaughter, Spain, 2017

**DOI:** 10.3389/fmicb.2019.02990

**Published:** 2020-01-28

**Authors:** Nerea García, Marta Hernández, Maialen Gutierrez-Boada, Antonio Valero, Alejandro Navarro, Milagros Muñoz-Chimeno, Alvaro Fernández-Manzano, Franco Matías Escobar, Irene Martínez, Carmen Bárcena, Sergio González, Ana Avellón, Jose M. Eiros, Gislaine Fongaro, Lucas Domínguez, Joaquín Goyache, David Rodríguez-Lázaro

**Affiliations:** ^1^VISAVET Health Surveillance Centre, Universidad Complutense Madrid, Madrid, Spain; ^2^Division of Microbiology, Department of Biotechnology and Food Science, Universidad de Burgos, Burgos, Spain; ^3^Department of Food Science and Technology, University of Córdoba, Córdoba, Spain; ^4^Laboratorio de Referencia e Investigación en Hepatitis Víricas, Centro Nacional de Microbiología, Instituto de Salud Carlos III, Madrid, Spain; ^5^Departamento de Microbiología e Inmunología, Universidad Nacional de Río Cuarto, Córdoba, Argentina; ^6^Department of Microbiology, Hospital Universitario Rio Hortega, Valladolid, Spain; ^7^Laboratory of Applied Virology, Universidade Federal de Santa Catarina, Florianópolis, Brazil; ^8^Department of Animal Health, Faculty of Veterinary, Universidad Complutense Madrid, Madrid, Spain

**Keywords:** hepatitis E virus, prevalence, seroprevalence, pigs, pork cuttings, pig organs, food safety, slaughterhouse

## Abstract

Zoonotic hepatitis E, mainly caused by hepatitis E virus (HEV) genotype (gt) 3, is a foodborne disease that has emerged in Europe in recent decades. The main animal reservoir for genotype 3 is domestic pigs. Pig liver and liver derivates are considered the major risk products, and studies focused on the presence of HEV in pig muscles are scarce. The objective of the present study was to evaluate the presence of HEV in different organs and tissues of 45 apparently healthy pigs from nine Spanish slaughterhouses (50% national production) that could enter into the food supply chain. Anti-HEV antibodies were evaluated in serum by an ELISA test. Ten samples from each animal were analyzed for the presence of HEV RNA by reverse transcription real-time PCR (RT-qPCR). The overall seroprevalence obtained was 73.3% (33/45). From the 450 samples analyzed, a total of 26 RT-qPCR positive samples were identified in the liver (7/45), feces (6/45), kidney (5/45), heart (4/45), serum (3/45), and diaphragm (1/45). This is the first report on detection of HEV RNA in kidney and heart samples of naturally infected pigs. HEV RNA detection was negative for rib, bacon, lean ham, and loin samples. These findings indicate that pig meat could be considered as a low risk material for foodborne HEV infection.

## Introduction

Hepatitis E virus (HEV) is a small non-enveloped positive sense single-stranded RNA virus classified in Hepeviridae family (Emerson and Purcell, 2003) and is the main cause of viral acute hepatitis in humans worldwide (EFSA, 2017). In developing countries, the virus is mainly transmitted through contaminated water, whereas in industrialized countries, sporadic cases are basically related to animals, and hepatitis E is currently considered an emerging zoonotic disease (EFSA, 2017). In Spain, anti-IgG-VHE prevalence ranges from 0.6 to 10% in the general population (Echevarria et al., 2015), whereas it could reach up to 19% in persons exposed to pigs (Galiana et al., 2008). Generally, hepatitis E is a self-limiting disease, but it can become chronic or cause a severe disease in immunocompromised patients or with previous liver or chronic diseases (Pavio et al., 2010).

Pigs are an important zoonotic source of HEV. The swine population is considered endemic for HEV-genotype 3 (gt3) in many European countries (Pavio et al., 2010). Human cases in which foodborne route was implicated have been increasing during the last decade (EFSA, 2017). Pigs are susceptible to infection, but they do not suffer clinical disease, so visual inspections are not valid to detect possible HEV infection in the necropsy or during slaughter (Meng et al., 1997; van der Poel et al., 2001). Thus, laboratory tests are necessary to determine the potential contamination of tissues, organs, muscles, and fluids, which could enter the food chain. The development of precise strategies for the prevention and control of HEV infection should be based on advances in the knowledge of source, epidemiology, and control methods. As pork products (including meat) are highly prevalent in European food markets, it is necessary to evaluate the potential risks they represent relatively to HEV infection of humans.

Pork meat is the most widely consumed meat type in the world, with 112. 472 thousand tons consumed in 2018 (United States Department of Agriculture Foreign Agricultural Service [USDA], 2019). In Europe, Spain is the second producer after Germany, with 19% of the total pork sector production (MAPAMA, 2018). Moreover, Spain exported 2,196,648 tons of pork products in 2018 (MAPAMA, 2018). HEV-gt3 is present in swine populations in different European countries and has been linked to cases of hepatitis E in several countries (Lapa et al., 2015). In Spain, HEV has been circulating in pig populations at least since the 1980s, reaching a farm seroprevalence up to 98% [95% confidence interval (CI) 96.1–99.9%] (de Deus et al., 2008; Seminati et al., 2008; Casas et al., 2009a; Jimenez de Oya et al., 2011). HEV prevalence in Spanish domestic pig serum samples was determined at 18.8% (64/341) (Jimenez de Oya et al., 2007).

The European Food Safety Authority (EFSA) has recommended integrated studies in the food chain to determine the potential risk of HEV in pork products (EFSA, 2017). Although some studies have evaluated HEV presence in pig liver, bile, feces, or serum in abattoirs, extensive data are lacking about the HEV presence in other organs or muscles that can enter the food chain. Consequently, the objective of the present work was to investigate the presence of HEV in pig products at the moment of slaughter, in an endemic country, to determine the potential risk of pork products, especially pork meat.

## Materials and Methods

### Sampling Strategy

A cross-sectional study on pigs being slaughtered between November and December 2017 was undertaken through a sampling strategy with a national coverage. Nine Spanish slaughterhouses were selected according to their slaughter capacity, which represents 50% of national pig production, and were located in different regions within the country. In each slaughterhouse, five animals (between 5 and 6 months old) were randomly selected. From each animal, 10 different samples were obtained: 10 ml of blood, 100–125 g feces, and approximately 25 g of heart, kidney, liver, ribs, bacon, diaphragm, lean ham (femoral biceps), and loin head. To avoid cross contamination, sterile scalpel blades and disposable material were used for each sample, and samples were taken with the appropriate hygienic precautions. Samples were refrigerated and sent to the laboratory in less than 8 h and frozen at −80°C until processing.

### Antibody Detection by ELISA

Once in the laboratory, blood was conserved at refrigeration temperature until the next day, when serum was obtained and stored at −20°C. Serum samples were tested for the presence of antibodies using the ID Screen Hepatitis E multi species indirect ELISA (IDvet, Montpellier, France) validated for swine based on recombinant gt3 capsid antigens. This ELISA kit detects IgG anti-HEV. Test procedures and interpretation of results were performed according to the manufacturer’s instructions. ELISA tests were repeated three times when the sample tested negative.

### Detection of HEV by Real-Time PCR

#### Sample Process Control Virus

A sample process control virus (SPCV) was added to each sample immediately before the start of the analysis. The SPCV was murine norovirus 1 (MNV-1) (Diez-Valcarce et al., 2011a), which had been propagated in RAW264.7 cells to a concentration of 10^7^ TCID_50_/ml, and a spike containing approximately 3 × 10^3^ TCID_50_ was added to each sample.

#### Virus Concentration and Nucleic Acid Extraction From Pork Organs and Cuttings

The meat samples (1 cm^3^ from three different locations) were collected and stored in a sterile plastic bag. The extraction procedure was based on a mechanical disruption of the tissues followed by a silica-membrane-based RNA extraction (Di Bartolo et al., 2012; Rodriguez-Lazaro et al., 2012). Briefly, each sample (approximately 1 g) was finely chopped using a sterile razor blade, and then 100 mg of homogenate was transferred into a Fast Prep tube containing 200 μl phosphate-buffered saline (PBS) and 2 g of sterile 1-mm zirconia beads (BioSpec Products, Inc., Bartlesville, OK, United States). Twenty microliters of MNV-1 (∼3 × 10^3^ TCID_50_) was added to each tube. The tube was then placed into a mechanical disruptor (FastPrep-24, MP Biomedicals, Santa Ana, CA, United States) and subjected to two cycles at a speed of 4 m × s^–1^ for 40 s. Afterward, 1 ml QIAzol (Qiagen, Hilden, Germany) was added to the tubes and vortexed 30 s, and the mixture was dispensed into a new Fast Prep tube and incubated 5 min at room temperature. At that point, 200 μl chloroform:isoamyl alcohol (24:1 Sigma Aldrich) was added, the mixture was vortexed 15 s and incubated 5 min at room temperature and then centrifuged at 10,000 × *g* for 15 min at 4°C. The resulting supernatant was used for immediate nucleic acid extraction using RNeasy Lipid Tissue Mini kit (Qiagen, Hilden, Germany) following manufacturer instructions, and the final 100 μl RNA extract was assayed immediately or stored at −80°C.

#### Virus Concentration and Nucleic Acid Isolation From Pork Feces and Serum

Two hundred fifty milligrams of samples were transferred to a 15-ml centrifuge tube and suspended in 2.25 ml of PBS containing gentamycin (10 mg/ml), and 20 μl of the SPCV (∼3 × 10^3^ TCID_50_) was added to the sample. The suspension was vortexed for 60–90 s and centrifuged at 3,000 × *g* for 15 min. For serum analysis, 20 μl of the SPCV (∼3 × 10^3^ TCID_50_) was added to 1 ml of blood, and the blood sample was centrifuged at 2,500 × *g* for 10 min. The supernatants from fecal or serum samples were then immediately used for nucleic acid isolation or stored at −80°C. Nucleic acids were extracted using a QIAamp viral RNA mini kit (Qiagen, Hilden, Germany) according to the manufacturer’s instructions. The final elution was performed twice with 50 μl elution buffer, resulting in a 100-μl nucleic acid extract. The nucleic acid extract was assayed immediately or stored at −80°C until analysis.

#### Virus Detection by RT-qPCR

The presence of the target virus (HEV) and the SPCV (MNV-1) was evaluated using reverse transcription real-time PCR (RT-qPCR). All reaction mixes included an internal amplification control (IAC), which was constructed as described by Diez-Valcarce et al., 2011a, b.

One-step duplex RT-qPCRs were performed using the oligonucleotides, controls, and conditions previously described (Diez-Valcarce et al., 2011b, 2012; Martinez-Martinez et al., 2011; Di Bartolo et al., 2012; Rodriguez-Lazaro et al., 2015). The thermocycling conditions varied slightly: 15 min at 50°C, 2 min at 95°C, followed by 45 cycles of 15 s at 95°C and 1 min at 60°C. All RT-qPCRs were conducted in a duplex format, targeting the specific viruses (HEV or MNV-1) with a FAM-labeled probe and the chimerical IAC using a VIC-labeled probe. All tests also included negative controls for viruses and for IACs.

#### Reporting and Interpretation of Data

For a proper interpretation of the results, four different signals were considered: (i) the target virus; (ii) the SPCV virus; (iii) the target IAC; (iv) the SPCV IAC (D’Agostino et al., 2011). When a PCR assay showed a Cq (quantification cycle) value ≤ 40, independently of the corresponding IAC Cq value, the result was interpreted as positive. When an assay showed a Cq value ≥ 40 with the corresponding IAC Cq value ≤ 40, the result was interpreted as negative. When both the target and its corresponding IAC showed Cq values ≥ 40, the reaction was considered to have failed. When at least one of the replicate HEV assays was positive, the sample was considered to be positive. In the absence of signals for SPCV and its IAC, the pre-amplification process (virus concentration and extraction steps) was concluded to have failed (D’Agostino et al., 2011). When signals for SPCV and its IAC and target IAC were present, the absence of target virus signal was conclusively considered a test negative result.

#### Extraction Efficiency

The extraction efficiency was calculated by comparing the Cq value of the sample containing the control (SPCV) with the Cq value of the SPCV alone, just spiked in the reagents used for concentration and extraction of the sample but without any food matrix, using the following formula: 2 ^(CqTNPC^ ^–Cqsample)^ × 100 (Diez-Valcarce et al., 2012). Efficiency results were classified as insufficient (extraction efficiency <5%), acceptable (5–25%), good (25–50%), and very good (>50%). Extraction efficiencies lower than 5% were not acceptable, and the pre-amplification process (virus concentration and extraction) of the given sample was repeated.

### HEV Genotyping

Positive samples for HEV were subjected to sequence analysis, partially amplifying and sequencing ORF2, as described previously (Munoz-Chimeno et al., 2016). Phylogenetic analyses were performed with the Mega 7.0 using the method neighbor-joining with 1,000 bootstrap.

### Statistical Analysis

Calculations for descriptive statistics were carried out using the WINPEPI (PEPI-for-Windows) computer programs for epidemiologist V.11.30 (Abramson, 2004). All data were compared using the χ^2^ test with 95% CIs, and a *p*-value < 0.05 was considered statistically significant. In addition, a generalized linear regression model with mixed effects (GLM) was performed considering the type of sample collected (loin head, heart, ribs, diaphragm, liver, liver exudate, lean ham, bacon, kidney, feces, and blood) and slaughterhouse (nine establishments from A to I) as explanatory variables against the binary response variable (i.e., detection of HEV by using RT-qPCR method). A backward selection method was chosen, and mean estimated parameters together with goodness-of-fit indices were obtained. The latter corresponded to the log likelihood (logL), Akaike Information Criterion (AIC), and Bayesian Information Criterion (BIC). The model structure was defined as:

$$\log\text{it}\,\left(\pi \right)=\log\left(\frac{\text{π}}{1-\pi }\right)=y_{i}={\text{β}}_{\text{o}}+\beta_{1}\cdot x_{i,1}+\ldots +{\text{β}}_{p-1}\cdot x_{i,p-1}+{\text{ε}}_{i}∼\text{Normal}\,\left(0,\sigma^{2}\right)$$

which models the log odds of probability of the presence of HEV RNA by RT-qPCR (*y*_*i*_) as a function of a set of explanatory variables (*x*_*i*_, _1_ … *x*_*i*_, *_*p*_*_–__1_). β_0_, β_1_, …β*_*p*_*_–__1_ are the unknown regression parameters, and σ^2^ the unknown (constant) error variance. The logit link function (logit π) models the log odds of the mean (π), assuming a binomial distribution of *y*_*i*_. The software R v.3.5.1^1^ was used, taking as a level of significance a *p*-value < 0.05.

## Results

### Detection of Anti-HEV Antibodies in Pig Sera

Thirty-three of the 45 pigs of the study showed IgG antibodies against HEV, which represents an overall seroprevalence of 73.3% (95% CI: 58.9–84.0). Seropositive animals were found in all of the nine slaughterhouses evaluated in this study.

### Efficiencies of HEV Nucleic Acid Extraction

The mean virus extraction efficiency of the process was 50.3% with a standard error of 1.83%. Values ranged from 2.26 to 98.4%. Overall, 14.6% of the samples showed acceptable extraction efficiency (5–25%), and 42.2 and 42.6% showed good (25–50%) and very good (>50%) extraction efficiencies, respectively.

### Detection of HEV RNA and Distribution Between the Type of Samples and Slaughterhouses

Table 1 summarizes the results obtained for the presence of HEV RNA in the 10 different types of samples tested in this study. Significant differences in the presence of HEV RNA were obtained (*p* < 0.001). Although only 26 out of 450 samples (5.78%; 95% CI: 3.97–8.33%) were positive by the HEV-specific RT-qPCR, those samples came from 20 pigs; that is, 20 pigs were positive for at least one of the 10 types of analyzed samples, which represents a 44.4% (95% CI: 30.9–58.8%) of the total of pigs tested (Table 2). However, only four pigs were HEV RNA-positive in two or more samples tested (one and three pigs with four and two HEV RNA-positive samples, respectively) (Table 2). Consequently, there were 16 animals (35.6%; 95% CI: 23.3–50.2%) with only one positive sample, three (6.67%; 95% CI: 2.29–17.9%) with two positive samples and one (2.22%; 95% CI: 0.39–11.6%) with four positive samples. In three of those four animals, one of the positive samples was feces (Table 2).

**TABLE 1:** Overall results of hepatitis E virus (HEV)-specific ELISA and RT-qPCR tests.

	**ELISA**					**HEV qPCR**						
		
	**Serum**	**Serum**	**Feces**	**Liver**	**Kidney**	**Heart**	**Diaphragm**	**Bacon**	**Loin head**	**Rib**	**Lean**	**Total**
Positive samples % (95% CI)	33 73.3% (58.9–84.0%)	3 6.7% (2.3–17.9%)	6 13.3% (6.3–26.2%)	7 15.6% (7.7–28.8%)	5 11.1% (4.9–23.9%)	4 8.9% (3.5–20.7%)	1 2.2% (0.4–11.6%)	0	0	0	0	26 5.8% (4.0–8.3%)
Cq values M ± ES	n.a.	36.0 ± 2.0	36.6 ± 1.9	36.6 ± 1.1	38.7 ± 0.6	38.7 ± 0.4	36.0	0	0	0	0	37.3 ± 0.6

**TABLE 2:** Distribution of positive samples for hepatitis E virus (HEV)-specific ELISA and RT-qPCR tests according to the type of samples.

**N. samples**	**ELISA**	**HEV qPCR**									
	**Serum**	**Serum**	**Feces**	**Liver**	**Kidney**	**Heart**	**Diaphragm**	**Bacon**	**Head loin**	**Rib**	**Lean**
1	−	−	−	+	−	−	−	−	−	−	−
1	−	−	−	−	+	−	−	−	−	−	−
1	−	+	+	+	−	−	+	−	−	−	−
1	+	+	−	−	−	−	−	−	−	−	−
1	+	+	−	−	−	+	−	−	−	−	−
1	+	−	+	−	−	−	−	−	−	−	−
1	+	−	+	+	−	−	−	−	−	−	−
1	+	−	+	−	+	−	−	−	−	−	−
2	−	−	+	−	−	−	−	−	−	−	−
3	+	−	−	−	+	−	−	−	−	−	−
3	+	−	−	−	−	+	−	−	−	−	−
4	+	−	−	+	−	−	−	−	−	−	−
7	−	−	−	−	−	−	−	−	−	−	−
18	+	−	−	−	−	−	−	−	−	−	−
Positive samples	33	3	6	7	5	4	1	0	0	0	0

The mean Cq values were very low (37.3 ± 0.6 SE) regardless the type of sample analyzed, ranging from 27.8 to 39.9. Interestingly, the distribution of positive samples varied according to the type of samples analyzed; whereas the muscle type samples were all negative (except for a single positive sample in the case of the diaphragm), the number of positive samples was significantly higher in the case of samples from organs (liver: *n* = 7, 15.5%; kidney: *n* = 5, 11.1%; and heart: *n* = 4, 8.89%) or from stool samples (*n* = 6, 13.3%) and serum (*n* = 3, 5.57%) (Table 1 and Supplementary Table 1). Regarding slaughterhouses (from A to I), all of them apart from E had HEV-positive samples. The highest prevalence corresponded to F (15.2%; 95% CI: 4.8–25.6%) with 7 out of 46 positive samples whereas for A, B, C, D, G, H, and I, average prevalence of HEV ranged from 3.9 to 7.8%. Although some variability was observed between slaughterhouses, differences were not statistically significant (*p* = 0.186).

### HEV Genotyping

**FIGURE 1:**
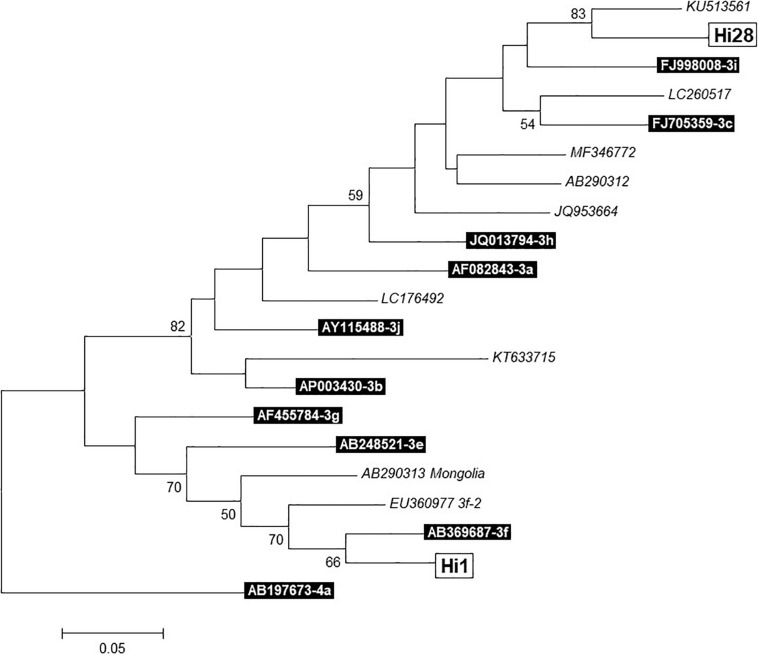
Phylogenetic tree of two sequences (Hi1 and Hi28) obtained from liver samples. Built with MEGA 7.0.

According to the mean Cq values, sequencing yield of the samples for HEV genotyping was low; however, two sequences were obtained (from liver samples of pigs 1 and 28). After phylogenetic analysis (Figure 1), Hi1 sequence was identified as genotype 3f, and Hi28 sequence showed a high identity with the KU513561 Spanish sequence, previously described in humans, which is pending of subtype assignment (Munoz-Chimeno et al., 2016).

### Correlation Between ELISA and RT-qPCR Results

Serology by ELISA and RT-qPCR for detection of HEV RNA in blood indicated that positive results obtained by both methods were not significantly correlated (Pearson χ^2^ = 0.073; *p* = 0.793). Similarly, seven animals tested negative for HEV detection both in ELISA and in the RT-qPCR, which indicates that only a 15.5% (95% CI: 7.75–28.7%) of the pigs had not been in contact with the HEV. Similarly, 18 pigs (40.0%; 95% CI: 27.0–54.5%) tested seropositive but were negative by the HEV-specific RT-qPCR (Table 2), which highlights that although the animals were not infectious at the moment of slaughter, they had been in contact with the virus previously. Fifteen pigs were seropositive and also tested positive by RT-qPCR in at least one of the samples analyzed (33.3%; 95% CI 21.3–47.9%); HEV RNA was detected in four liver, four kidney, and four heart samples and in three feces (Table 2); 12 animals tested RNA-positive in one of the samples, and the three remaining pigs were positive in two samples (feces and liver; feces and kidney; serum and heart). Finally, five animals tested seronegative but were positive by RT-qPCR in at least one of the samples analyzed for each animal (11.1%; 95% CI: 4.84–23.5%). From those animals, two excreted HEV in feces (RNA-positive), although they tested negative by both RT-qPCR and ELISA in the rest of the samples, two tested RNA-positive only in liver or kidney, respectively, and finally one seronegative animal was RNA-positive in diaphragm, liver, fecal, and serum samples.

### GLM Model for Detection of HEV RNA

The estimations obtained by the GLM model are represented in Table 3. Results from loin head samples together with those from slaughterhouse E were considered controls because all samples were negative. Considering the control group, the odds of having one HEV RNA-positive sample were calculated for the interactions between type of sample and slaughterhouse. Overall, all single factors were considered non-significant terms in the GLM model (*p* > 0.05). Furthermore, the odds ratio (OR) was estimated, quantifying the strength of the association between individual explanatory variables (type of sample and slaughterhouse) and the response variable (presence of HEV RNA). The strongest association was found for the kidney samples from slaughterhouse B (OR = 1.64, 95% CI: 1.11–2.44), meaning that the odds of finding one HEV RNA-positive sample was 64% higher than the control group for this combination. Other significant interactions were obtained for liver and kidney samples from slaughterhouses F, C, and G and for feces and blood samples from slaughterhouses A and F (Table 3). The odds of finding one HEV RNA-positive sample were 49% higher than the control group for these combinations.

**TABLE 3:** Estimations of the generalized linear regression model with fixed effects for the presence of hepatitis E virus (HEV) RNA by RT-qPCR as a function of the type of sample and slaughterhouse.

**Variable**	**Estimate**	**Std. Error**	***t* value**	***p*-value**	**Odds Ratio**
*Faces × Slaughterhouse A*	0.400	0.195	2	0.0404*	1.49 (95% CI: 1.01–2.18)
*Kidney × Slaughterhouse B*	0.500	0.201	2	0.0130*	1.64 (95% CI: 1.11–2.44)
*Kidney × Slaughterhouse C*	0.400	0.195	2	0.0404*	1.49 (95% CI: 1.01–2.18)
*Liver × Slaughterhouse F*	0.400	0.195	2	0.0404*	1.49 (95% CI: 1.01–2.18)
*Blood × Slaughterhouse F*	0.400	0.195	2	0.0404*	1.49 (95% CI: 1.01–2.18)
*Liver × Slaughterhouse G*	0.400	0.195	2	0.0404*	1.49 (95% CI: 1.01–2.18)

* Non-significant in the GLM model (*p* > 0.05).

The goodness of fit indices AIC, logL, and BIC were estimated as −14.31, 131.32, and 403.90, respectively. Regarding model predictions, considering a cutoff value of 0.10 for the probability of having one HEV RNA-positive sample in the collected samples, 84.06% of the cases observed as negative were correctly predicted by the model, whereas 15.94% of observed negative cases were misclassified as positive (fail-safe). However, all positive cases were correctly classified by the model as such, thus indicating the high discriminatory power of the GLM model according to the studied factors.

## Discussion

In this study, anti-HEV antibodies detection in serum and molecular analyses of 10 different samples (serum, feces, liver, diaphragm, kidney, heart, bacon, head of loin, rib, and lean) was performed in 45 apparently healthy pigs from different farms and collected from nine slaughterhouses geographically widespread in Spain. The overall seroprevalence obtained by an ELISA test, 73.3% (33/45), was not unexpected because a high anti-HEV antibody prevalence has been observed in an apparently healthy swine population since the 1980s in Spain (Casas et al., 2009b, 2011; Jimenez de Oya et al., 2011). This result is also in agreement with reports from a variety of European countries, as the seroprevalence described in farmed pigs ranged from 30 to 98% (Salines et al., 2017). Besides, in swine abattoirs, results are similar as for example a 59% (55.5–61.4%) of HEV seroprevalence was recently found in France (Feurer et al., 2018). Interestingly, in our study, a total of seven animals resulted negative for HEV detection by both ELISA and RT-qPCR tests, which indicates that only a maximum of 15.56% (95% CI: 7.75–28.7) was not previously in contact or infected by HEV. Not surprisingly, the presence of anti-HEV IgG in serum was higher (73.3%) than the presence of the virus detected by RT-qPCR (44.4%) in concordance with previous studies (Di Bartolo et al., 2011), and more than a half of the seropositive animals (18/33) were negative to the RT-qPCR (54.5%, 95% CI: 37.9–70.1), indicating a previous contact with HEV, but not current infection. This fact can be explained by the decrease in the prevalence of HEV RNA detection between 3 and 6 months described by previous studies (Casas et al., 2011). Five seronegative animals were positive for the RT-qPCR in different samples analyzed (41.7%, 95% CI: 19.3–68.1). Similar results were observed in the study of Di Bartolo et al. (2011) in which 4/6 seronegative animals were positive for the presence of viral RNA in bile, feces, and/or liver samples (Di Bartolo et al., 2011). Among them, two animals excreted the HEV by feces, but they tested negative in other samples, which could indicate the pass of the virus through the intestinal system after oral ingestion without any replication of the virus, as other authors have suggested (Di Bartolo et al., 2011). Besides, one animal was positive only in liver and another one only positive in the kidney. Finally, one seronegative animal was HEV RNA-positive in diaphragm, feces, liver, and serum samples, although only IgG antibodies have been detected. Absence of anti-HEV antibodies (IgG, IgM, and IgA) in pigs with HEV RNA in muscle has been described before in an experimental study of coinfection with porcine reproductive and respiratory syndrome virus (PRRSV), hypothesizing that the cause could be HEV replication in muscle cells favored by PRRSV or an interaction between heparin sulfate expressed at the surface of muscle cells with HEV particles during a long-term viremia (Salines et al., 2019a). This also could be explained by recent viral infection in which no immune response is detectable or due to a chronic infection in which antibodies may disappear because they do not persist for a long time, as other studies had demonstrated (Kanai et al., 2010).

Twenty animals tested HEV RNA-positive for at least one of the 10 samples analyzed, which indicated that in a high percentage of the pigs tested (44.4%, 95% CI: 30.9–58.8), HEV had disseminated through the organism, similar to the results previously obtained by Di Bartolo et al. (2012), with 38% (15/39) positive samples in a Spanish slaughterhouse. This is also in accordance with other authors who also reported the presence of HEV RNA in many different samples, such as lymph nodes, bladder, liver, bile, or tonsils collected from pigs in abattoirs (Leblanc et al., 2010; Raspor Lainscek et al., 2017; Feurer et al., 2018). Similarly, a total of 26 samples (5.78%) tested RT-qPCR-positive for HEV in our study: liver (*n* = 7; 15.5%), feces (*n* = 6; 13.3%), kidney (*n* = 5; 11.1%), heart (*n* = 4; 8.89%), serum (*n* = 3; 6.67), and diaphragm (*n* = 1; 2.22%) (Table 1 and Supplementary Table 1). Cq values ranged from 27.8 to 39.5, indicating a different viral load, although no association was observed between the Cq value and the type of sample (Table 2). As expected, liver was the most frequently positive sample identified with 15.6% (95% CI: 7.75–28.8), as it is the target organ for HEV replication (Lee et al., 2009). In previous studies, liver, also with the bile, had been described as the highest infected tissue (de Deus et al., 2007; Leblanc et al., 2010). In contrast, other studies describe feces as the sample with the highest prevalence for HEV presence (Di Bartolo et al., 2012; Raspor Lainscek et al., 2017). The liver positivity rate evidenced in our study (15.6%) is similar to the one obtained in Italy (20.8%) (Di Bartolo et al., 2011). However, it is higher than results obtained by other researchers in different regions, including Europe, Africa, and South America, and summarized in 2017 by Salines et al. (2017) with a mean of 5.3% (from 0.8% in Cameroon to 10% in Canada). This must be explained by the fact that some risk factors have been associated with the presence of HEV RNA in pig liver such as coinfection with PRRSV (Salines et al., 2019b), age, genetic background, or lack of hygienic measures (Walachowski et al., 2014). Nevertheless, it must be taken into account that in the present study, as in most of the published works, the presence of the HEV genetic material (HEV RNA) had been demonstrated, but the potential infectivity was not evaluated due to the difficulty of systems, which determine the viability of the virus (reliable cell culture systems or animal experimental models). However, HEV-contaminated pig liver can enter the food chain with the consequent risks demonstrated by numerous studies. In the review of Salines et al. (2017), nine different studies conducted on market pork products (raw livers, sausages, paté, etc.) were analyzed, and contamination with HEV ranged from 1 to 50% depending on the product analyzed and on the country where the survey was conducted. The highest prevalence was in products made with raw liver such as figatelli from France (Pavio et al., 2014), confirming previous studies that indicated that liver is a risk product for HEV infection, especially if it is consumed raw or undercooked, not only from pigs but also from wild boars and deer (Yazaki et al., 2003; Mizuo et al., 2005; Feagins et al., 2007). Our findings highlight that liver could be contaminated with HEV and could represent a risk for the consumer if is not well-cooked and confirms that pig liver and liver-made products must be controlled.

Similarly, six of 45 animals were shedding HEV in feces at the time of slaughterhouse, reaching a positivity of 13.3% (95% CI: 6.26–26.2), similar (6/43) to that observed in France (Leblanc et al., 2010) but lower than those observed in other countries 20–32% in Portugal (Berto et al., 2012), 21.5% in the United Kingdom (McCreary et al., 2008), 33.3% in Italy (Di Bartolo et al., 2011), or 55% in Denmark (Breum et al., 2010). It is important to highlight that as pigs are excreting the HEV in the feces, it is indispensable to optimize hygienic measures in abattoirs to avoid cross contamination with materials that could enter the food chain.

A very interesting finding in our study was that only one muscle sample (1/225; 0.44%) was HEV RNA-positive. This animal was also RT-qPCR-positive in feces, liver, and serum but was negative to the ELISA test. HEV presence in pig muscle has been demonstrated in experimental studies (Williams et al., 2001; Bouwknegt et al., 2009; Salines et al., 2019a), but few studies have been performed to establish the presence of HEV in pig muscles in naturally infected animals. Our findings are in agreement with the studies conducted in French and Canadian slaughterhouses in which the HEV seroprevalence was high, but the virus was not found in muscle samples (Leblanc et al., 2010; Feurer et al., 2018) and with a longitudinal study performed in Denmark (Krog et al., 2019). Also, one study analyzing pork products at the market failed in the detection of HEV RNA in pork chops and fresh sausages (Boxman et al., 2019). Some studies have detected HEV RNA in meat samples, but the prevalence was very low; one study performed on lingual muscle revealed a prevalence of 3 and 6% in Czechia and in Italy, respectively, whereas no positive samples were found in Spain (Di Bartolo et al., 2012). Cross contamination with feces, bile, or utensils cannot be ruled out (Di Bartolo et al., 2012).

Studies linking boar meat (Matsuda et al., 2003; Tamada et al., 2004; Li et al., 2005; Masuda et al., 2005; Wichmann et al., 2008; Rivero-Juarez et al., 2017) and also deer meat (Tei et al., 2003, 2004) with hepatitis E cases have been described since many years ago. Reports were mainly from Asia probably due to consumption habits (eating raw or undercook food). However, cases directly associated with pork meat are limited to the study of Deest et al. (2007), who reported the case of two patients who had eaten undercooked pig meat 4 weeks before suffering hepatitis E (Deest et al., 2007). Besides, in a case control study in Germany, raw pig meat and sausage were not associated with HEV cases (Wichmann et al., 2008). Although pork meat is not usually consumed raw, it is more probably that pig meat is not frequently contaminated with HEV or the viral load is too low to cause infection, confirmed by previous studies (Leblanc et al., 2010; Feurer et al., 2018) and the present study. The few studies previously performed on pig muscle have failed in the detection of HEV (Leblanc et al., 2010; Crossan et al., 2015; Krog et al., 2019).

This study is the first description of HEV RNA in kidney or heart in naturally infected pigs with five and four positive for heart and four kidney samples, respectively. These organs are not usually evaluated for the presence of HEV in pigs, and only two experimental studies have tested these organs (Williams et al., 2001; Bouwknegt et al., 2009). Extrahepatic dissemination in pigs was confirmed (Williams et al., 2001; Bouwknegt et al., 2009; Thiry et al., 2016), although the viral load was lower in those localizations (Krog et al., 2019). This fact is corroborated in the present study as the mean Cq values were higher in heart and kidney (Table 1 and Supplementary Table 1). To explain the HEV distribution to other organs or tissues other than the liver, different causes could be invoked, such as other concomitant diseases (e.g., PRRSV), which could influence the infection dynamics (Salines et al., 2019b).

## Conclusion

In conclusion, our findings highlight that the Spanish pigs were frequently in contact with HEV previously (high seropositivity rate). In addition, and at the moment of slaughter, HEV could be present in pig liver, the virus could be being actively excreted (HEV RNA found in feces), and even in some cases, pigs could display viremia (HEV found in serum). Unfortunately, we only obtained two HEV sequences that limit gaining a better understanding of HEV transmission between pigs and humans. However, our results demonstrate that HEV appears to be almost always absent in different pork muscles, so pork meat could be considered as a low risk material for HEV infections *via* foodborne route. To confirm this hypothesis, future studies that include a larger number of animals are needed.

## Data Availability Statement

The datasets generated for this study are available on request to the corresponding authors.

## Author Contributions

NG, JG, DR-L, LD, and MH designed the study. CB collected the samples at slaughterhouses. MG-B, FE, IM, AF-M, and AN performed the laboratory analysis. SG and AA designed the tables and figures. AA and MM-C performed the genotype analysis, including ORF amplification, sequencing, and phylogenetic analysis. AV, GF, and JE performed statistical analysis of the data obtained. NG and DR-L analyzed the data. NG drafted the first version of the manuscript. DR-L and SG modified and adapted the draft version, which was subsequently revised by MH and JG.

## Conflict of Interest

The authors declare that the research was conducted in the absence of any commercial or financial relationships that could be construed as a potential conflict of interest.

## Footnotes

1 www.cran.rproject.org
